# An Interpretable Four-Gene Cell-Cycle Signature Links Malignant Progression to Adverse Survival in Adult Primary Diffuse Glioma: A CGGA Transcriptome Study

**DOI:** 10.3390/genes17070833

**Published:** 2026-07-21

**Authors:** Hongkai Jia, Nan Pu, Chunyan Tian

**Affiliations:** 1 Renal Division, Peking University Institute of Nephrology, Peking University First Hospital & Key Laboratory of Renal Disease, Ministry of Health of China & Key Laboratory of Chronic Kidney Disease Prevention and Treatment (Peking University), Ministry of Education of China & Research Units of Diagnosis and Treatment of Immune-Mediated Kidney Diseases, Chinese Academy of Medical Sciences & Beijing Key Laboratory of Precision Medicine and New-Drug/Equipment Development for Severe Kidney Disease, Beijing 100034, China; 2 College of Bioengineering, Beijing Polytechnic University, Beijing 100176, China; 3 Department of Ultrasound Medicine, People’s Hospital of Yubei District, Chongqing 401120, China

**Keywords:** adult diffuse glioma, Chinese Glioma Genome Atlas, machine learning, transcriptomics, survival signature, CDK1, CCNB2, CDCA3, PTTG1

## Abstract

Background: Adult diffuse gliomas are biologically heterogeneous and clinically lethal. We evaluated whether a compact transcriptomic program could summarize malignant progression and adverse survival. Methods: We analyzed adult primary WHO grades II–IV gliomas from the CGGA mRNAseq_693 cohort and used CGGA mRNAseq_325 as an independent validation cohort. *CDK1*, *CCNB2*, *CDCA3*, and *PTTG1* were prioritized through a transparent, literature-informed mitotic-gene framework. Four classifiers were evaluated by five-fold cross-validation repeated 20 times, with all preprocessing restricted to each training fold. A training-defined four-gene score was assessed by Kaplan–Meier and Cox models. Results: The primary cohort contained 415 tumors, including 396 with evaluable survival. We identified 773 differentially expressed genes between WHO grade IV and WHO grades II/III tumors, and all four candidate genes increased monotonically with grade. In repeated cross-validation, the SVM achieved the highest mean AUROC of 0.793 ± 0.048 (95% CI 0.784–0.802), with average precision of 0.701 ± 0.063. In the independent CGGA mRNAseq_325 cohort (*n* = 226), logistic regression achieved an AUROC of 0.792. The four-gene score and a broader four-marker proliferation index showed similar discrimination, supporting the interpretation of the signature as a compact proliferation-axis proxy. The training-set median cut-off was −0.153. The score remained associated with shorter survival after adjustment for age, grade, and *IDH* status in the primary cohort (*n* = 359; HR = 1.28, 95% CI 1.10–1.49; *p* = 0.002) and in the independent cohort (*n* = 217; HR = 1.58, 95% CI 1.25–2.00; *p* < 0.001). Conclusions: The four-gene score captures grade-associated proliferative biology and reproducible survival risk across two CGGA cohorts. It is a research biomarker rather than a standalone diagnostic or clinical decision tool.

## 1. Introduction

Adult-type diffuse gliomas are now classified according to histology-integrated molecular criteria, with the principal adult entities including oligodendroglioma (*IDH*-mutant and 1p/19q-codeleted), astrocytoma (*IDH*-mutant), and glioblastoma (*IDH*-wildtype) [1]. Among these tumors, glioblastoma remains the most lethal despite surgery, radiotherapy, and temozolomide-based treatment, with a median overall survival of approximately 15 months in routine practice [2]. At the same time, clinically relevant heterogeneity exists across and within diffuse glioma subtypes, making prognostic assessment and therapeutic stratification difficult [3].

High-dimensional transcriptomic resources have opened a path toward more precise biological classification. However, many published signatures rely on unstable gene lists, mixed tumor populations, limited transparency, or insufficient validation. A recent systematic review and meta-analysis of machine-learning and deep-learning applications in glioblastoma concluded that these approaches are promising, but also emphasized persistent problems of data heterogeneity, variable reporting standards, and the need for stronger validation frameworks [4].

The Chinese Glioma Genome Atlas (CGGA) provides a clinically annotated resource for glioma transcriptomics and malignant-progression research [5,6]. Because the dataset contains grade information, molecular markers, and overall survival, it is well-suited for asking a clinically important question: Can a small, interpretable gene set summarize the transcriptional shift from lower-grade diffuse glioma to WHO grade IV disease while also retaining prognostic information?

Cell-cycle dysregulation is a biologically plausible axis for such a signature. Aberrant activation of cyclin-dependent kinases and mitotic regulators is a canonical hallmark of cancer biology [7]. *CDK1* is a master regulator of mitotic entry and has been implicated in glioblastoma biology [8]. *CCNB2*, a cyclin B family member, contributes to G2/M transition and has already been associated with prognosis in glioma [9]. *CDCA3* regulates mitotic progression and has recently been proposed as a glioma biomarker [10]. *PTTG1* acts as securin, promotes genomic instability and invasive behavior, and has been connected to adverse glioma biology in several studies [11,12,13]. Temozolomide-related regulation of *CDK1* has also been reported in glioblastoma [14].

In the present study, we performed a rigorously curated re-analysis of CGGA mRNAseq_693 data restricted to adult primary diffuse glioma. We first established a transcriptome-wide landscape of malignant progression, then focused on a compact four-gene mitotic signature consisting of *CDK1*, *CCNB2*, *CDCA3*, and *PTTG1*. Using interpretable machine learning and survival modeling, we evaluated whether this four-gene program could summarize WHO grade IV-associated transcriptional progression, reflect aggressive molecular contexts, and stratify overall survival. Because the candidate genes were prioritized using grade-associated biology, classification analyses were interpreted as internal confirmation of the discriminatory information carried by the program, not as evidence of an independently discovered diagnostic classifier.

## 2. Materials and Methods

### 2.1. Public Dataset and Cohort Curation

This study analyzed publicly available, de-identified transcriptomic and clinical data from the CGGA mRNAseq_693 cohort (20200506 release) and used the independent CGGA mRNAseq_325 cohort from the same release for external validation [5,6]. Gene-level RSEM abundance values and matched clinical annotations were downloaded from CGGA. Expression values were transformed as log2(RSEM + 1), and expression and clinical identifiers were matched exactly. Identical restrictions to adults aged 18 years or older, primary tumors, and WHO grades II–IV yielded 415 cases in mRNAseq_693 and 226 cases in mRNAseq_325. No cross-cohort batch correction was applied; models were fitted in mRNAseq_693 and evaluated without refitting in mRNAseq_325.

To maximize clinical comparability and avoid mixing pediatric or recurrent disease biology into the primary analysis, we restricted the study population to adults aged 18 years or older, primary tumors, and WHO grades II–IV. This yielded 415 adult primary diffuse gliomas. For survival analysis, we further required non-missing overall survival (OS) and vital-status annotation and excluded OS shorter than 30 days to reduce the influence of peri-diagnostic bias, resulting in a final survival cohort of 396 cases. Clinical covariates were used as recorded in the CGGA file. Cases lacking a required endpoint or covariate were omitted from the corresponding model, and missing annotations were not imputed. Because the study used public de-identified data only, no additional institutional ethics approval or patient consent was required for this secondary analysis.

### 2.2. Transcriptome-Wide Screening and Candidate-Gene Prioritization

To characterize malignant progression at the transcriptome level, we compared adult primary WHO grade IV tumors against the combined WHO grades II/III group. For each gene, mean expression difference, Welch t statistic, and two-sided *p* value were computed on the log2-transformed matrix. Benjamini–Hochberg adjustment was used to control the false discovery rate (FDR). Differentially expressed genes (DEGs) were defined primarily as FDR < 0.01 with an absolute mean difference >1.

Candidate-gene prioritization followed an explicit two-stage framework. First, the transcriptome-wide WHO grade IV versus WHO grades II/III comparison was used to define the broader malignant-progression landscape. Second, a focused four-gene set (*CDK1*, *CCNB2*, *CDCA3*, and *PTTG1*) was prioritized for detailed modeling according to four prespecified interpretability criteria: significant grade association in the CGGA screen, monotonic increase from WHO grade II to WHO grade III to WHO grade IV, coherent biology around mitotic control or securin function, and feasibility as a compact transcript-level panel. *CDK1*, *CCNB2*, and *PTTG1* met the stringent DEG effect-size and FDR thresholds. *CDCA3* showed a slightly smaller mean difference than the DEG effect-size cut-off but was retained because it showed a highly significant monotonic grade-associated pattern and has prior literature support as a mitotic regulator and glioma biomarker [10]. Thus, the four-gene set was a hypothesis-driven, biologically anchored panel rather than an exhaustive high-dimensional prognostic search [8,9,10,11,12,13,14].

Supplementary Table S1 reports the 25 leading WHO grade IV-upregulated genes together with the four panel genes, grade-specific means, mean difference, Welch *p* value, FDR, monotonic grade trend, DEG-threshold status, panel status, selection rationale, and biological annotation.

### 2.3. Four-Gene Score and Molecular-Context Analyses

A composite four-gene score was defined as the mean of gene-wise z-scores for *CDK1*, *CCNB2*, *CDCA3*, and *PTTG1*. Expression differences across WHO grades were assessed by Kruskal–Wallis tests, while binary comparisons (primary versus recurrent and *IDH*-mutant versus *IDH*-wildtype) used Mann–Whitney U tests. Spearman correlation coefficients were calculated between the four candidate genes and representative proliferation markers (*MKI67*, *TOP2A*, *AURKA*, and *PCNA*).

For direct comparison with generic proliferation measures, we evaluated *MKI67* alone, *TOP2A* alone, and the mean z-score of *MKI67*, *TOP2A*, *AURKA*, and *PCNA*. Each score was standardized using training-fold parameters for repeated cross-validation and using mRNAseq_693 parameters for independent mRNAseq_325 validation. Classification comparisons used one-predictor logistic regression, and survival comparisons used Cox models adjusted for age, WHO grade, and *IDH* status.

Because the clinical file did not directly encode all final WHO 2021 integrated diagnoses, we generated exploratory molecular-context groups using available markers: *IDH*-mutant/1p/19q-codeleted tumors were labeled oligodendroglioma-like, *IDH*-mutant/1p/19q-non-codeleted tumors were labeled astrocytoma-like, and *IDH*-wildtype WHO grade IV tumors were labeled GBM-like. These groupings were used descriptively to contextualize the four-gene score rather than to replace formal integrated diagnosis. This distinction is important because some molecular features required for full WHO 2021 classification, such as *TERT* promoter mutation, *EGFR* amplification, and combined whole chromosome 7 gain/chromosome 10 loss, were not fully represented in the curated analysis table [1].

### 2.4. Interpretable Machine-Learning Models for Grade IV Discrimination

**Figure 1:**
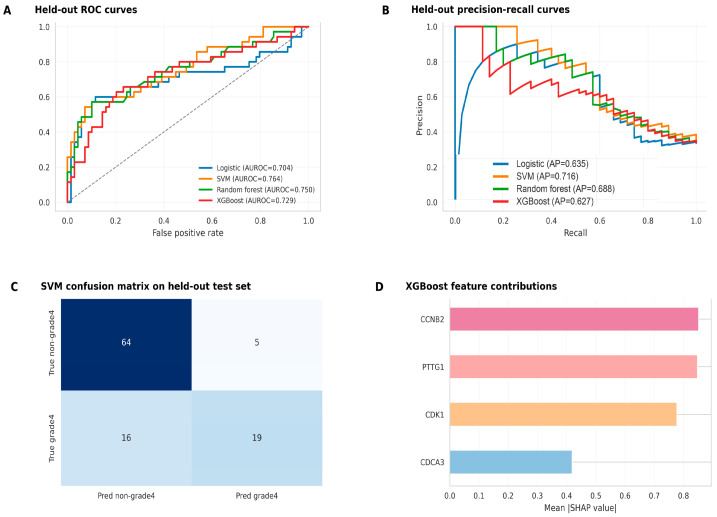
Interpretable machine-learning discrimination of WHO grade IV tumors. (**A**) Held-out receiver operating characteristic curves for logistic regression, SVM, random forest, and XGBoost. (**B**) Held-out precision–recall curves. (**C**) Confusion matrix of the best-performing SVM model on the test set. (**D**) XGBoost feature ranking based on mean absolute SHAP values. SHAP was used to interpret the XGBoost model and should not be read as a direct explanation of the SVM classifier.

Machine-learning classification was limited to *CDK1*, *CCNB2*, *CDCA3*, and *PTTG1*. Logistic regression used L2 regularization with C = 1.0 and the liblinear solver. The SVM used an RBF kernel with C = 1.0 and gamma = scale. The random forest used 500 trees and square-root feature sampling. XGBoost used 200 trees, maximum depth 2, learning rate 0.05, subsample 0.8, and column subsample 0.8. Standardization was performed within each training fold for logistic regression and SVM. Five-fold stratified cross-validation was repeated 20 times with random seed 20260703. AUROC, average precision, accuracy, sensitivity, and specificity were summarized across 100 validation folds as mean ± SD and 95% CI. Final models were fitted to all 415 mRNAseq_693 cases and evaluated without refitting in the independent mRNAseq_325 cohort (*n* = 226). The original 75:25 held-out analysis was retained as a secondary visualization in Figure 1.

To facilitate biological interpretation of nonlinear model behavior, SHAP (Shapley additive explanations) values were computed for the XGBoost model [15]. Mean absolute SHAP values were used to rank the relative contribution of each gene to grade IV classification in XGBoost. This analysis was considered complementary to the main performance comparison and was not interpreted as directly explaining the best-performing SVM classifier.

### 2.5. Survival Analyses and Statistical Framework

For prognostic evaluation, the mRNAseq_693 survival cohort was divided 70:30 with stratification by WHO grade and event status using random seed 20260703. Gene means and standard deviations were estimated only in the training set and applied unchanged to the held-out test set and the independent mRNAseq_325 cohort. The four-gene score was the mean of the four training-standardized expression values. The training-set median score (−0.153) was prespecified as the high-risk versus low-risk cut-off and was applied without optimization to all validation analyses. Kaplan–Meier curves, numbers at risk, and log-rank tests were reported. Tertile and quartile sensitivity analyses compared the highest and lowest score groups.

Univariable and multivariable Cox proportional hazards models used the continuous four-gene score. The main complete-case model included age, WHO grade as a numeric step, and IDH status coded as IDH-mutant = 0 and IDH-wildtype = 1. Schoenfeld residual tests assessed proportional hazards. Because the global test indicated non-proportionality, a sensitivity model stratified jointly by WHO grade and *IDH* status was fitted. An extended complete-case model additionally included sex, 1p/19q codeletion, *MGMT* promoter methylation, radiotherapy, and chemotherapy status. Harrell C-index values compared the score-only, clinical, and integrated models.

Analyses used Python 3.9.6, numpy 1.26.4, pandas 2.3.3, scipy 1.13.1, scikit-learn 1.6.1, xgboost 2.1.4, lifelines 0.30.0, matplotlib 3.9.4, and seaborn 0.13.2. All tests were two-sided with alpha = 0.05.

## 3. Results

### 3.1. Cohort Characteristics and Transcriptome-Wide Landscape of Malignant Progression

The curated cohort consisted of 415 adult primary diffuse gliomas with a median age of 43 (35–53) years and a male predominance (239 (57.6%)). Grade distribution covered the disease spectrum, comprising 134 WHO grade II tumors, 142 WHO grade III tumors, and 139 WHO grade IV tumors. After excluding patients without usable survival annotation or with OS shorter than 30 days, 396 cases remained for prognostic analysis. Baseline clinical and molecular features, including missing molecular annotations, are summarized in Table 1.

**Figure 2:**
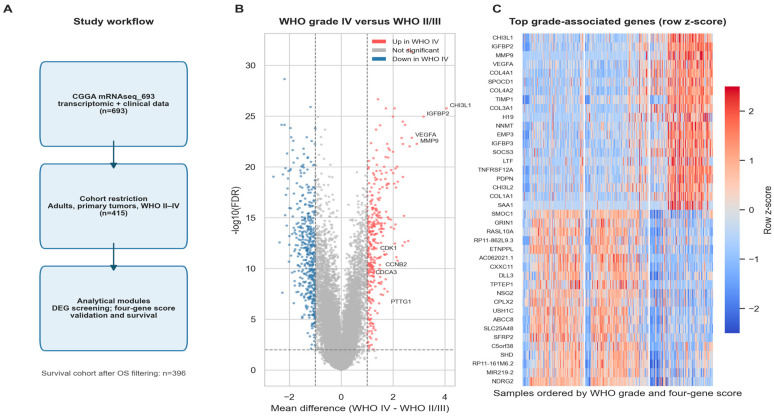
Study design and transcriptome-wide landscape of malignant progression. (**A**) Workflow summarizing CGGA data curation, cohort restriction, and downstream analysis modules; arrows indicate the sequential order of the workflow steps. (**B**) Volcano plot comparing adult primary WHO grade IV tumors with WHO grades II/III tumors; red points indicate genes upregulated in WHO grade IV and blue points indicate genes downregulated in WHO grade IV using FDR < 0.01 and absolute mean difference >1. (**C**) Heatmap of the top 20 upregulated and top 20 downregulated genes after row-wise z-scoring, with columns ordered by WHO grade and four-gene score.

When WHO grade IV tumors were compared with the combined WHO grades II/III group, 773 genes met the prespecified DEG threshold (FDR < 0.01 and absolute mean difference > 1), including 328 genes upregulated in WHO grade IV and 445 genes downregulated in WHO grade IV (Figure 2B,C). The volcano plot and heatmap revealed a strong transcriptomic gradient spanning lower-grade tumors to grade IV disease, consistent with coordinated biological remodeling rather than isolated single-gene effects. Among the upregulated components, several mitotic regulators were conspicuous, supporting a focused analysis of the cell-cycle axis.

### 3.2. CDK1, CCNB2, CDCA3 and PTTG1 Increase with Grade and Aggressive Molecular Context

**Figure 3:**
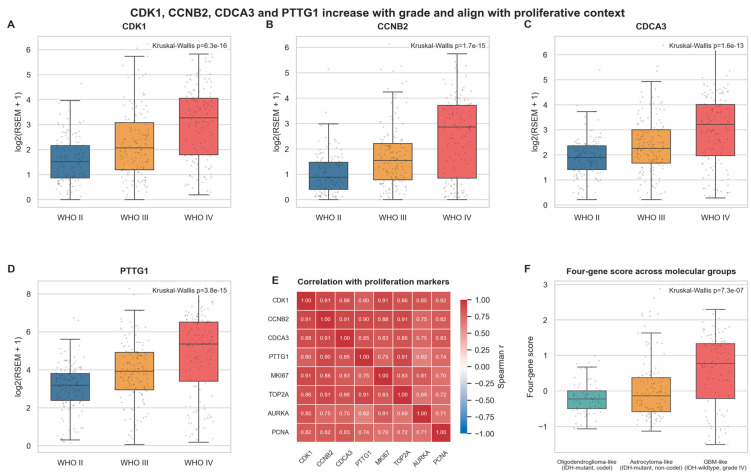
*CDK1*/*CCNB2*/*CDCA3*/*PTTG1* expression increases with grade and molecular aggressiveness. (**A**–**D**) Boxplots showing grade-wise expression distributions for *CDK1*, *CCNB2*, *CDCA3*, and *PTTG1*. (**E**) Spearman correlation matrix linking the four-gene signature to representative proliferation markers. (**F**) Distribution of the composite four-gene score across exploratory molecular-context groups: oligodendroglioma-like, *IDH*-mutant/1p/19q-codeleted; astrocytoma-like, *IDH*-mutant/1p/19q-non-codeleted; GBM-like, *IDH*-wildtype WHO grade IV.

All four candidate genes displayed progressively higher expression from WHO grade II to WHO grade III to WHO grade IV, with Kruskal–Wallis *p* < 0.001 for each gene (Figure 3A–D). *CDK1*, *CCNB2*, and *PTTG1* also exceeded the stringent DEG effect-size threshold, whereas *CDCA3* showed a slightly smaller mean difference but preserved a highly significant and monotonic grade-dependent pattern. This combination of strong statistical support, monotonic grade association, and coherent mitotic biology justified retaining all four genes in the compact signature.

The four-gene program aligned closely with proliferative biology: Spearman correlations between signature genes and the proliferation markers *MKI67*, *TOP2A*, *AURKA*, and *PCNA* ranged from 0.62 to 0.91 (Figure 3E). At the composite-score level, the four-gene score differed across exploratory molecular-context groups (Kruskal–Wallis *p* < 0.001), with the highest values in the GBM-like group (Figure 3F). The score was also significantly higher in recurrent than in primary adult gliomas (*p* < 0.001), and higher in *IDH*-wildtype than in *IDH*-mutant tumors (*p* < 0.001), indicating that the signature tracks molecular aggressiveness beyond conventional grade alone.

The four-gene score and generic proliferation measures showed closely similar discrimination. In repeated cross-validation using one-predictor logistic models, mean AUROC was 0.715 for the four-gene score, 0.708 for *MKI67*, 0.711 for *TOP2A*, and 0.722 for the four-marker proliferation index. In the independent cohort, AUROC values were 0.813, 0.758, 0.785, and 0.812, respectively (Supplementary Table S4). These results indicate that the four-gene score is a compact proliferation-axis proxy rather than a glioma-specific discriminator that clearly outperforms established proliferation measures.

### 3.3. Interpretable Machine-Learning Discrimination of WHO Grade IV Tumors

Using the four-gene feature set alone, the SVM achieved the highest repeated cross-validation AUROC, with a mean of 0.793 ± 0.048 (95% CI 0.784–0.802), average precision of 0.701 ± 0.063, accuracy of 0.767 ± 0.032, sensitivity of 0.483 ± 0.074, and specificity of 0.911 ± 0.036 across 100 validation folds (Table 2). XGBoost showed a similar mean AUROC of 0.786, followed by random forest at 0.764 and logistic regression at 0.718. In the independent mRNAseq_325 cohort (*n* = 226), logistic regression achieved the highest AUROC of 0.792, while XGBoost achieved the highest average precision of 0.700 (Supplementary Table S3). The original held-out SVM result (AUROC = 0.764) is retained in Figure 1 as a secondary single-split visualization.

Feature attribution analysis further supported the internal coherence of the XGBoost model. In the XGBoost SHAP interpretation, *CCNB2* and *PTTG1* contributed the highest mean absolute SHAP values, followed by *CDK1* and *CDCA3* (Figure 1D). The SVM confusion matrix showed high specificity for non-grade-IV tumors but limited sensitivity for WHO grade IV detection: 19 of 35 grade-IV cases were correctly identified, and 16 were missed, whereas 64 of 69 non-grade-IV cases were correctly classified. This reinforces that the four-gene panel should be viewed as a parsimonious biological discriminator rather than a stand-alone clinical diagnostic classifier.

### 3.4. Four-Gene Score Stratifies Overall Survival in Training, Test, and Clinically Relevant Subgroups

**Figure 4:**
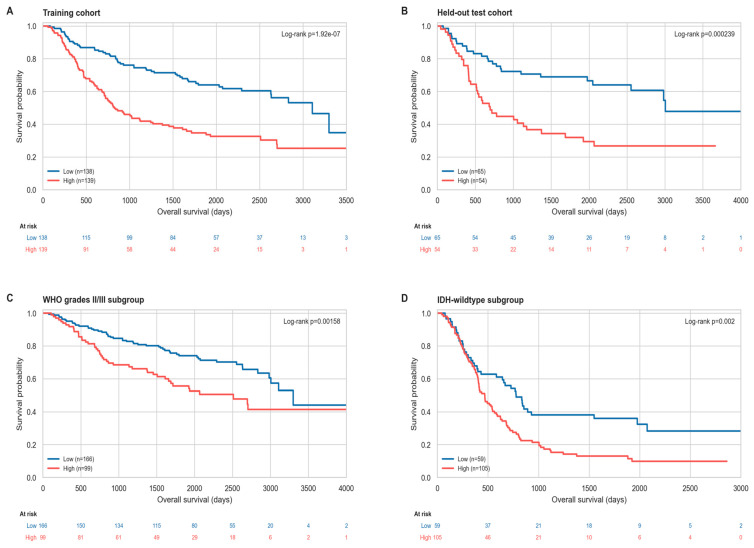
Prognostic stratification of the four-gene score. (**A**) Kaplan–Meier analysis in the training cohort using the predefined median score cut-off. (**B**) Kaplan–Meier analysis in the held-out test cohort using the same cut-off. (**C**) Survival stratification within the WHO grades II/III subgroup. (**D**) Survival stratification within the *IDH*-wildtype subgroup.

Within the mRNAseq_693 survival cohort (*n* = 396; 207 events), the training-set median four-gene score of −0.153 separated high-risk and low-risk groups. Survival differed in the training cohort (*n* = 277; log-rank *p* < 0.001), held-out test cohort (*n* = 119; *p* < 0.001), WHO grades II/III subgroup (*n* = 265; *p* = 0.002), and *IDH*-wildtype subgroup (*n* = 164; *p* = 0.002) (Figure 4). In the independent mRNAseq_325 survival cohort (*n* = 218; 136 events), the same mRNAseq_693-derived scaling parameters and cut-off also separated survival (log-rank *p* < 0.001; Supplementary Table S10).

Cut-off sensitivity analyses supported the continuous-score result. After adjustment for age, WHO grade, and *IDH* status, the highest versus lowest tertile was associated with HR = 1.97 (95% CI 1.34–2.88; *p* < 0.001), and the highest versus lowest quartile was associated with HR = 1.71 (95% CI 1.09–2.68; *p* = 0.019) (Supplementary Table S8).

**Figure 5:**
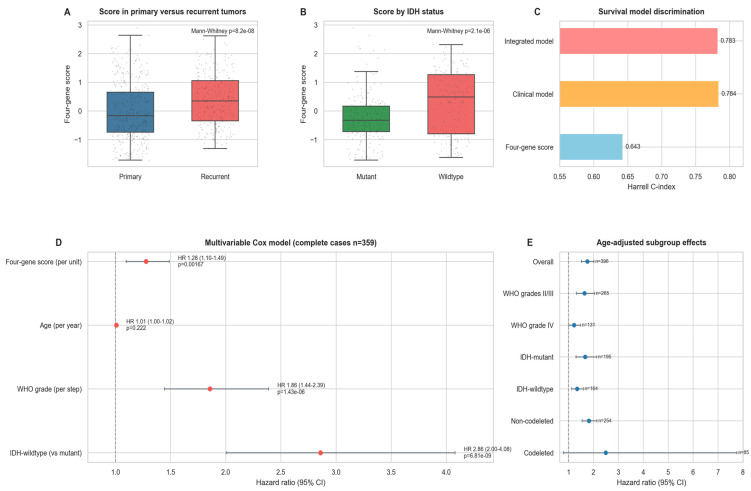
Clinical integration and subgroup robustness of the four-gene score. (**A**) Training-standardized score in primary versus recurrent adult glioma. (**B**) Score according to *IDH* status in adult primary tumors. (**C**) Harrell C-index values for the score-only, clinical, and integrated models. (**D**) The main multivariable Cox model was applied to 359 complete cases. (**E**) Age-adjusted subgroup hazard ratios with subgroup sample sizes.

The prognostic effect remained visible in clinically important subsets. The training-defined cut-off separated survival among WHO grades II/III tumors (log-rank *p* = 0.002; Figure 4C) and *IDH*-wildtype tumors (*p* = 0.002; Figure 4D). Age-adjusted continuous-score Cox models were significant in the overall, WHO grades II/III, WHO grade IV, *IDH*-mutant, *IDH*-wildtype, and non-codeleted groups; the codeleted subgroup was not significant and contained only 14 events (Figure 5E; Supplementary Table S9).

### 3.5. Independent Prognostic Value and Clinical Integration

In the main complete-case multivariable analysis (*n* = 359; 197 events), each one-unit increase in the training-standardized four-gene score was associated with a 28% higher hazard of death after adjustment for age, WHO grade, and *IDH* status (HR = 1.28, 95% CI 1.10–1.49; *p* = 0.002) (Table 3; Figure 5D). The score was independently validated in mRNAseq_325 (*n* = 217 complete cases; HR = 1.58, 95% CI 1.25–2.00; *p* < 0.001).

The score-only model achieved a Harrell C-index of 0.643. The clinical model achieved 0.784, and the integrated model achieved 0.783, indicating no incremental discrimination beyond the clinical variables (Figure 5C). Schoenfeld-residual tests indicated non-proportionality for the four-gene score (*p* = 0.042) and *IDH* status (*p* < 0.001), with a global *p* = 0.002. In a sensitivity model stratified jointly by WHO grade and *IDH* status, the score remained associated with survival (HR = 1.23, 95% CI 1.06–1.43; *p* = 0.007). In the extended model containing sex, 1p/19q status, *MGMT* methylation, radiotherapy, and chemotherapy (*n* = 240; 136 events), the score also remained significant (HR = 1.54, 95% CI 1.23–1.93; *p* < 0.001) (Supplementary Table S7).

## 4. Discussion

This study provides a curated, interpretable transcriptomic analysis of adult primary diffuse glioma using the CGGA mRNAseq_693 cohort. By restricting the analysis to adult primary tumors, we reduced biological mixing introduced by pediatric disease biology and recurrence-associated treatment effects. By focusing on a compact four-gene set rather than a large opaque signature, we preserved biological interpretability and improved the feasibility of downstream assay development. The grade comparison, molecular-context analysis, classification benchmarking, and survival modeling converged on a consistent biological axis: progressive activation of cell-cycle machinery as tumors approach an aggressive WHO grade IV and *IDH*-wildtype phenotype. Within the framework of the WHO 2021 integrated classification, such compact transcript-level signatures are best viewed as complementary research markers that may refine biological risk interpretation alongside established molecular taxonomy, not as replacements for formal diagnosis [1,5,6].

The biological coherence of the signature strengthens its plausibility. *CDK1* is a central catalytic node of G2/M transition and remains one of the most compelling cell-cycle targets in cancer biology [7,8]. Recent mechanistic work in glioblastoma further showed that OTUD4 can promote tumor progression by deubiquitinating *CDK1* and activating MAPK signaling, directly linking *CDK1* stabilization to malignant behavior in glioma cells [16]. *CCNB2* provides an essential cyclin partner for mitotic entry and has already been associated with adverse outcomes in glioma [9]. *CDCA3* regulates mitotic progression and has been proposed as a biomarker of glioma malignancy, while broader CDCA-family analyses suggest that these programs may also track therapeutic vulnerabilities such as rapamycin sensitivity [10,17]. *PTTG1* connects proliferation, chromosomal instability, invasion, and poor prognosis in glioma [11,12,13]. Taken together, these observations support the interpretation that the composite score captures a coordinated mitotic state characterized by proliferative competence and genomic-risk biology.

From a data-mining perspective, the present work addresses an important translational tension in the machine-learning literature. Systematic synthesis indicates that ML and deep-learning models in glioblastoma are promising but remain limited by heterogeneous cohorts, variable endpoints, inadequate external validation, and inconsistent reporting practices [4]. The current frontier is increasingly multimodal and explicitly interpretable. A biologically interpretable multi-task deep-learning pipeline recently demonstrated concurrent prediction of molecular alterations, grade, and prognosis in glioma patients [18]. In 2025, GlioMT used an interpretable multimodal transformer for adult-type diffuse glioma grading and molecular-subtype prediction [19], whereas GlioSurv extended multimodal transformer modeling to individualized survival prediction and achieved strong concordance across cohorts [20]. At the systems level, multimodal fusion of radio-pathology and proteogenomics has also revealed integrated glioma subtypes with prognostic and therapeutic implications [21]. AI-assisted liquid-biopsy and extracellular-vesicle spectroscopy work further illustrates that biomarker discovery is expanding beyond tissue transcriptomics alone [22]. Against this background, our study intentionally occupies a different niche: it does not attempt to maximize predictive ceiling through high-dimensional multimodal fusion, but instead evaluates a minimalist transcriptomic signature that remains transparent, assayable, and suitable for future validation in routine molecular workflows.

The survival analysis further clarifies the role of this signature. The four-gene score remained significant after adjustment for age, WHO grade, and IDH status in mRNAseq_693 and was independently validated in mRNAseq_325. However, the clinical model C-index was 0.784, and the integrated model C-index was 0.783. Statistical significance, therefore, did not translate into incremental discrimination beyond routine clinical variables. The strongest current interpretation is that the score reflects a proliferative and biologically aggressive tumor state rather than a clinically deployable risk rule.

The multi-endpoint consistency of the findings supports biological coherence. The four genes increased with grade, aligned with canonical proliferation markers, contributed to grade-IV discrimination, and retained prognostic value across two CGGA cohorts. Nevertheless, direct comparison showed that the four-gene score and a broader *MKI67*/*TOP2A*/*AURKA*/*PCNA* index had nearly identical external classification AUROCs (0.813 and 0.812) and similar clinical-model C-indices. The panel should therefore be interpreted as a compact, assayable representation of the proliferation axis rather than as evidence of superiority over generic proliferation measures.

Several limitations should be acknowledged. First, although the principal results were independently validated in CGGA mRNAseq_325, both cohorts originate from the same atlas and release framework. Validation in TCGA, other institutions, assay platforms, and prospective series remains necessary. Second, the same grade-associated contrast informed candidate prioritization and grade-IV classification. The classification analysis, therefore, confirms the information carried by a hypothesis-driven proliferative panel rather than constituting independent diagnostic discovery. Third, the SVM retained limited sensitivity despite high specificity, which precludes stand-alone diagnostic use.

Additional limitations relate to clinical annotation and biology. The WHO 2021 integrated classification could only be partially approximated because not all required molecular features were available in the curated working table. Several clinically relevant covariates, including extent of resection, *MGMT* promoter methylation, performance status, tumor location, radiotherapy, temozolomide or chemotherapy exposure, and detailed treatment course, were not incorporated into the main multivariable model because they were unavailable or insufficiently curated for the present analysis. Bulk transcriptomic data also reflect mixed tumor, immune, stromal, endothelial, necrotic, and hypoxic components; therefore, the four-gene score may be influenced by tumor purity or cell-state composition rather than exclusively tumor-intrinsic proliferation. Finally, the biological role of the four-gene program was inferred from expression patterns and the literature support, not from new wet-lab perturbation experiments.

Pathway enrichment and tumor-purity deconvolution were not added because the present revision focused on prespecified validation, prognostic sensitivity analyses, and transparent comparison with established proliferation measures. The absence of matched purity estimates and cell-resolved data remains an explicit limitation, and mechanistic claims were avoided.

Future work should therefore test the four-gene score across independent CGGA/TCGA-style resources, evaluate qPCR and immunohistochemical assay reproducibility, perform mechanistic perturbation studies in glioma models, and determine whether multimodal augmentation with MRI, digital pathology, proteomics, methylation, CNV, spatial transcriptomics, liquid biopsy, or extracellular-vesicle-derived signals can improve performance without sacrificing interpretability [20,21,22].

## 5. Conclusions

A four-gene cell-cycle score composed of *CDK1*, *CCNB2*, *CDCA3*, and *PTTG1* captures grade-associated proliferative biology and reproducible adverse survival across the CGGA mRNAseq_693 and independent mRNAseq_325 cohorts. Its discrimination is similar to that of a broader proliferation index, and it adds no measurable C-index improvement beyond core clinical variables. The score is best viewed as a compact research biomarker, not a stand-alone diagnostic, prognostic, or treatment-selection tool. Validation across independent institutions, platforms, and prospective cohorts remains required.

## Figures and Tables

**Table 1:** Baseline characteristics of the adult primary diffuse glioma cohort.

Characteristic	Value
Total adult primary diffuse gliomas	415
Age, median (IQR)	43 (35–53)
Male, *n* (%)	239 (57.6%)
Female, *n* (%)	176 (42.4%)
WHO grade II, *n* (%)	134 (32.3%)
WHO grade III, *n* (%)	142 (34.2%)
WHO grade IV, *n* (%)	139 (33.5%)
*IDH*-mutant, *n* (%)	207 (49.9%)
*IDH*-wildtype, *n* (%)	169 (40.7%)
1p/19q codel, *n* (%)	88 (21.2%)
1p/19q non-codel, *n* (%)	267 (64.3%)
GBM histology, *n* (%)	139 (33.5%)
Astrocytoma histology, *n* (%)	82 (19.8%)
Anaplastic astrocytoma histology, *n* (%)	81 (19.5%)
Oligodendroglioma histology, *n* (%)	44 (10.6%)
Anaplastic oligodendroglioma histology, *n* (%)	45 (10.8%)
*IDH* status missing, *n* (%)	39 (9.4%)
1p/19q status missing, *n* (%)	60 (14.5%)

Note: Percentages are calculated within the 415 adult primary diffuse gliomas. Molecular percentages do not sum to 100% because some cases had missing annotation; *IDH* status was missing in 39 cases (9.4%), and 1p/19q status was missing in 60 cases (14.5%). IQR, interquartile range. Table shading is used for formatting only.

**Table 2:** Repeated cross-validation and independent classification performance of four machine-learning models built from the four-gene panel.

Model	AUROC Mean ± SD [95% CI]	Average Precision Mean ± SD [95% CI]	Accuracy Mean ± SD [95% CI]	Sensitivity Mean ± SD [95% CI]	Specificity Mean ± SD [95% CI]	Independent CGGA325 AUROC
Logistic regression	0.718 ± 0.054 [0.707–0.728]	0.609 ± 0.074 [0.595–0.624]	0.760 ± 0.033 [0.753–0.766]	0.443 ± 0.083 [0.427–0.459]	0.919 ± 0.037 [0.912–0.926]	0.792
SVM	0.793 ± 0.048 [0.784–0.802]	0.701 ± 0.063 [0.688–0.713]	0.767 ± 0.032 [0.761–0.774]	0.483 ± 0.074 [0.468–0.498]	0.911 ± 0.036 [0.903–0.918]	0.769
Random forest	0.764 ± 0.048 [0.755–0.774]	0.673 ± 0.063 [0.661–0.685]	0.746 ± 0.039 [0.738–0.753]	0.540 ± 0.085 [0.524–0.557]	0.849 ± 0.051 [0.839–0.859]	0.775
XGBoost	0.786 ± 0.046 [0.776–0.795]	0.690 ± 0.062 [0.678–0.702]	0.747 ± 0.037 [0.740–0.754]	0.556 ± 0.087 [0.539–0.574]	0.843 ± 0.049 [0.834–0.853]	0.772

Note: Repeated internal validation used five-fold stratified cross-validation repeated 20 times (100 validation folds). Values are mean ± SD [95% CI]. Independent validation used 226 adult primary WHO grades II–IV tumors from CGGA mRNAseq_325; full external accuracy, sensitivity, specificity, and average precision are reported in Supplementary Table S3. Table shading is used for formatting only.

**Table 3:** Multivariable Cox proportional hazards model in adult primary diffuse glioma.

Variable	Hazard Ratio (95% CI)	*p* Value
Four-gene score (per unit)	1.28 (1.10–1.49)	0.002
Age (per year)	1.01 (1.00–1.02)	0.222
WHO grade (per step)	1.86 (1.44–2.39)	<0.001
*IDH*-wildtype (vs. *IDH*-mutant)	2.86 (2.00–4.08)	<0.001

Note: The four-gene score used training-set means and standard deviations. WHO grade was modeled numerically as a grade step, and *IDH* status was coded as *IDH*-mutant = 0 and *IDH*-wildtype = 1. The main Cox model included 359 complete cases and 197 events. Table shading is used for formatting only.

## Data Availability

The CGGA mRNAseq_693 and mRNAseq_325 expression matrices and matched clinical annotations analyzed in this study are publicly available from the Chinese Glioma Genome Atlas (CGGA; 20200506 release) and are described in references [5,6]. File names, source URLs, release identifiers, download date, and SHA256 checksums are provided in Supplementary Table S11. No original sequencing data were generated. The complete Python analysis and figure-generation script, dependency specification, and data-provenance instructions are provided as Supplementary File S2.

## Supplementary Materials

The following supporting information can be downloaded at: https://www.mdpi.com/article/10.3390/genes17070833/s1, Supplementary File S1 contains Tables S1–S12, including candidate-gene transparency, repeated cross-validation, independent validation, comparator, proportional-hazards, sensitivity, provenance, and software-version results. Supplementary File S2 contains the complete Python analysis and figure-generation code with its dependency specification.

## Abbreviations

AP	average precision
AUROC	area under the receiver operating characteristic curve
CGGA	Chinese Glioma Genome Atlas
DEG	differentially expressed gene
FDR	false discovery rate
GBM	glioblastoma
HR	hazard ratio
*IDH*	isocitrate dehydrogenase
OS	overall survival
SHAP	Shapley additive explanations
SVM	support vector machine
WHO	World Health Organization
